# Screening for monoclonal gammopathies of undetermined significance: A prospective study

**DOI:** 10.4102/jphia.v16i1.714

**Published:** 2025-03-08

**Authors:** Aissam El Maataoui, Sofia Farhat

**Affiliations:** 1 Department of Clinical Chemistry, Faculty of Medicine and Pharmacy, Ibn Zohr University, Agadir, Morocco

**Keywords:** pesticide applicators, monoclonal gammopathy of undetermined signification, cancer, Morocco, epidemiology

## Abstract

**Background:**

Several studies have reported an increase in the incidence of multiple myeloma among farmers following pesticide use.

**Aim:**

This study aimed to seek an association between pesticide exposure and monoclonal gammopathy of undetermined significance (MGUS) in young pesticide applicators.

**Setting:**

The setting for this study was the Souss Massa region of the Kingdom of Morocco.

**Methods:**

We conducted a case-control study among 239 young male pesticide applicators with known exposure to pesticides (male pesticide applicators = exposed group) and 157 males with no direct exposure to pesticides (unexposed group). Serum protein electrophoresis was performed on all sera, and when monoclonal proteins were detected, they were characterised by serum protein immunofixation electrophoresis.

**Results:**

We found that the prevalence of MGUS was significantly higher in the exposed group older than 40 years 4.03% (95% confidence interval [CI]: 1.49–9.62) compared with the control group 0.91% (95% CI: 0.16–4.97), with no cases reported before this age. However, the odds ratio did not reach statistical significance 3.33 (95% CI: 0.39–28.78), which can be explained by the size of the population and the mean age ± s.d. of the pesticide applicators of 39.54 ± 11.51. In contrast, the international studies found the same results with all recruited patients over 50 years. This selection was made at the beginning of the study. Monoclonal proteins in the exposed group were characterised as follows: immunoglobulin Gλ 3 (1.25%), IgGκ 1 (0.41%) and biclonal IgGκ + IgGκ 1 (0.41%).

**Conclusion:**

We recommend mandatory screening for monoclonal gammopathy in pesticide applicators over the age of 40 years. Further studies are needed to investigate the association between pesticide molecules and MGUS.

**Contribution:**

The results of this study can be used by the Ministry of Health and the Ministry of Agriculture to provide baseline data to help develop appropriate prevention measures and awareness programmes against the misuse of pesticides.

## Introduction

Multiple myeloma (MM) is a clonal plasma cells neoplasm characterised by established hypercalcaemia, renal failure, anaemia, lytic bone lesions (CRAB) features and three specific biomarkers: clone bone marrow plasma cells ≥ 60%, serum-free light chain ratio ≥ 100 and more than one focal lesion on magnetic resonance imaging (MRI).^1^ It accounts for 1% of all cancers worldwide, with an age-adjusted annual incidence of 4 cases per 100 000 people in the United States.^2^ In a Moroccan study of 261 patients, 52.77% were found to have MM,^3^ and in Algeria, MM was diagnosed in 55.20% of 2121 subjects.^4^ It is worth observing that the Moroccan and Algerian studies discussed next were hospital-based studies, and the prevalence of MM was so high because the patients of those studies were diagnosed at a late stage. None of the studies in the African population had a screening for monoclonal gammopathies in the exposed populations.

Multiple myeloma is the late stage of non-immunoglobulin M monoclonal gammopathy of undetermined significance (MGUS) or premalignant lesions. It is an asymptomatic disease defined by serum monoclonal protein < 3 g/dL, bone marrow clonal plasma cells < 10%, and failure to fulfil CRAB criteria.^1^ Monoclonal gammopathy of undetermined significance has a prevalence of 3.2% in the Caucasian population, which may be higher in African-American and obese populations.^4,5^ Notably, MGUS is asymptomatic and affects more than 3% of patients over the age of 50.^6^ The established risk factors for monoclonal gammopathies in general are sex (male), age, obesity, and race (African-American).^7^

Epidemiologic studies have demonstrated the adverse effects of pesticide exposure on human organs.^8^ The most frequently used synthetic insecticides are also more dangerous.^8^ Global reports demonstrate that farmers and farm workers are more susceptible than the general population to digestive, neurological, respiratory, retinal, and reproductive disorders because of their higher exposure to pesticides.^9,10^ However, there are discrepancies in research findings studying the association between pesticide exposure and cancer. Several studies have confirmed that the overall incidence of cancer in farm workers and pesticide applicators remains lower than expected compared to the general population,^11 12^ while other studies have found that agricultural activities and pesticide exposure are associated with the occurrence of monoclonal gammopathies, and agricultural workers have a significantly higher risk of MM.^13,14^ In a prospective study conducted in the United States of America on cancer incidence among private pesticide applicators in the Agricultural Health Study (AHS), the authors found a 1.34 (95% confidence interval [CI]: 0.97–1.81) risk of MM compared with the general population rates.^15^ Because common single nucleotide polymorphisms at 3p22.1 (rs1052501), 6p21.33 (rs2285803), 7p15.3 (rs4487645), and 17p11.2 (rs4273077) loci have been shown to influence the risk of developing MM and MGUS, it would be interesting to look for an association between pesticide exposure and these single-nucleotide polymorphisms (SNPs). Genetic variants such as cytochrome P450 family 1 subfamily B member 1 may be associated with the risk of developing MM in individuals exposed to exogenous chemicals.^16,17^

The main objective of this study is to seek an association between pesticide exposure and MGUS in young pesticide applicators.

## Research methods and design

### Study design and setting

This study used the guidelines of the Strengthening the Reporting of Observational Studies in Epidemiology (STROBE) statement. The objective of this study is to examine the potential correlation between pesticide use and MGUS. To this end, we have selected two groups for comparison: pesticide applicators whose primary responsibility is the preparation, treatment, and storage of pesticides, who are likely to have a high level of exposure, and other farmers who are not exposed to pesticides. This was a case-control study of male pesticide applicator and control groups. A case-control design was chosen because, firstly, MGUS is a relatively rare disease, making case-control studies more efficient for studying risk factors. Secondly, they are inexpensive, quick and easy to conduct. Thirdly, a case-control study allows for the development of prevention strategies for pesticide applicators.

### Participants

All participants were Moroccans and the sample size was calculated (*n*_0_ = 164) using the sample size formula in Equation 1:


$$n=z^{2}\times p(q)/d^{2}.$$ [Eqn 1]

where:

*n* = sample size

*z* = level of confidence according to the standard normal distribution (for a level of confidence of 95%, *z* = 1.96, for a level of confidence of 99%, *z* = 2.575)

*p* = Prevalence of MGUS in the Moroccan studies

*d* = tolerated margin of error (e.g. we want to know the real proportion within 5%).

This study enrolled 239 male pesticide applicators representing the exposure group and 157 men representing the non-exposure group working in the same geographic area. We chose to recruit pesticide applicators from among agricultural workers who have a proven occupational exposure to pesticides. The control group was selected from the male farmers residing and employed in the same rural agricultural area and time period as the cases. They were of the same age group as the pesticide applicators and had no history of occupational exposure to pesticides. There were no gender differences between the control and case groups (all males).

Women were excluded from the study because of the insufficient number in the exposure group (only 3 women); the patients who refused to provide informed consent to participate to the study or with a prior history of haematological malignancies were also excluded (1 patient).

### Data collection

To standardise the data collection, a questionnaire was used. The questionnaire requested information on the demographic characteristics (i.e., age, gender, education level …), the past medical history of pesticide applicators (i.e., allergies, family history of cancer, infections, asthma …). Some questions to measure the exposition to the pesticides (i.e., use of fall protection equipment, day per year of treatment with pesticides …) and cancer in first-degree relatives. The questionnaire was assessed by general practitioners and occupational physicians working at the farms. The participant’s responses were coded to remove the risk of error. To test the clarity and the outputs before the use of this instrument, the questionnaire was tested on 10 pesticide applicators.

The questionnaire was fulfilled by a doctor participating in the study to avoid bias. The questions were about medical and family history of cancer, workplace, job description, work under greenhouses or in open fields, the annual exposition, and the use of protective equipment. As a supplement material to this article, you will find the three lists of pesticides used in the tomatoes, citrus and red fruits crops. Some pesticides on the lists showed a positive significant association with MM (Captan, Pyrethrin) in some American and Canadian studies.^16^ All participants signed an informed consent document. The ethics committee at the faculty of medicine and pharmacy in Marrakech approved the study.

### Laboratory investigations

Blood samples were collected in tubes containing a clot activator. Quality controls, including normal and pathological controls, were performed before the reception of the samples. Serum protein electrophoresis (SPE) was performed at the reception of the samples in two different laboratories using Capillary electrophoresis Flex Sebia^®^. Total protein assay was performed on the Biosystem^®^ BA 400 analyser.

The results of the capillary electrophoresis were inspected by two clinical chemists of the two laboratories. Samples with a discrete or localised band on the electrophoresis were subjected to a serum protein immunofixation electrophoresis, using the serum immunofixation kit Sebia^®^ to characterise the immunoglobulin isotype. To confirm the diagnosis of MGUS in each patient with the presence of monoclonal protein in his serum, calcium, phosphate and creatinine were measured.

### Statistical analysis

Frequency and percentage were calculated to describe categorical variables. Quantitative variable results are presented as mean ± standard deviation (s.d.). To control for potential confounders, each case was individually matched with a control based on geographic area, agricultural work, potential pesticide exposure, and age, as these factors are known to influence the risk of developing MGUS. The normal distribution of continuous variables was verified by the Kolmogorov-Smirnov test. Analysis of variance (ANOVA) and Student’s t-test were used to compare continuous variables between groups. We computed the odds ratio (OR) and 95% CIs for associations of MGUS with the use of the pesticides. A *p* < 0.05 was considered statistically significant. For the statistical processing of the data, each participant’s responses were coded and validated to remove the risk of error, then Excel 2007 (Microsoft Corp., Redmond, WA) and Statistical Package for Social Sciences (SPSS) 13.0 (SPSS Inc., Chicago, IL) were used.

### Ethical considerations

Men who volunteered to participate in the study were enrolled after giving informed and written consent. Ethical clearance to conduct this study was obtained from the University Hospital Ethics Committee of the Faculty of Medicine and Pharmacy in Marrakech, Morocco on 13 July 2020. Before the start of data collection, informed consent was requested and obtained from each person included.

## Results

All the pesticide applicators were Caucasian Moroccans living in the Souss-Massa region. The exposed group and the control group have been stratified by age group with an interval of 10 years. This approach was selected because the prevalence of monoclonal gammopathies is known to increase from the age of 50 years and to continue to rise with age. A large prospective screening study conducted in Olmsted County, Minnesota subsequently estimated a prevalence of 3% and 5.3% among individuals aged > 50 and > 70 years, respectively.^18^

Table 1 shows that the pesticide applicators enrolled in the study were young with a mean age ± s.d. of 39.54 ± 11.51, with a large proportion of participants between 30 and 50 years of age. Table 2 shows the description of the study population; the overall prevalence rate of MGUS in pesticide applicators aged from 18 to 65 years was 2.1% [95% CI: 0.7% – 4.8%] (Table 2). However, when the study population was stratified by age, no cases of MGUS were detected in 115 subjects younger than 40 years old, whereas the prevalence rates in the 40 years to 50 years and 50 years to 60 years age groups, the prevalence rates were 4.29% and 4.25%, respectively. Thus, age 40 years and older was associated with a high risk of monoclonal gammopathy in the exposed group with an odds ratio (OR) of 1.04% (95% CI: 1.005% – 1.077%). In addition, all patients with monoclonal gammopathy in the exposed group were non-obese, had no past medical history, and no family history of cancer, never attended school, and the percentage of illiterates in the exposed group was 50.2%; they had no past medical history and no cancer in first-degree relatives. All patients with monoclonal gammopathy in the exposed group are pesticide applicators working in open fields with more than 52 h of pesticide application per year. Finally, the majority of cases of MGUS (four cases) were found in patients who worked in citrus groves (Table 3).

**TABLE 1:** Age distribution of the pesticide applicators.

Variable	*n*	Age (m ± s.d.)	95% CI
All the patients	239	39.54 ± 11.51	38.081–40.999
≥ 19–20	5	18.8 ± 0.45	18.406–19.194
20–30	52	24.90 ± 3.39	23.97–25.82
30–40	58	34.56 ± 2.8	33.929–35.371
40–50	70	44.11 ± 2.9	43.431–44.789
50–60	47	53.82 ± 2.74	53.037–54.603
≥ 61	7	62.71 ± 2.56	60.814–64.6

Note: *p*-value < 0.05 indicates a statistically significant result.

m ± s.d., mean ± standard deviation; CI, confidence interval.

**TABLE 2:** Comparison of the age and the prevalence between normal patients and patients with MGUS.

Variable	MGUS (*n* = 5)	Normal (*n* = 234)	*p*
Comparison of the age between normal patients and patients with MGUS m ± s.d. (95% CI)	49 ± 3.146(95% CI: 40.26–57.73)	39.34 ± 0.753(95% CI: 37.85–40.82)	0.035
Prevalence of MGUS in all age groups (18–65 years) (%)	5 (2.1%)	-	-

Note: Student’s *t*-test, *p*-value < 0.05 indicates a statistically significant result.

MGUS, monoclonal gammopathy of undetermined signification; CI, confidence intervals; m ± s.d., mean ± standard deviation.

**TABLE 3:** Description of the study population with MGUS in the exposed group.

Variable	MGUS (*n* = 5)		Normal (*n* = 234)	Odds ratio	95% CI
	*n*	%			
**Prevalence of MGUS by age group (years)**					
≥ 19	0	0	-	-	-
20-30	0	0	-	-	-
30-40	0	0	-	-	-
40-50	3	4.29	-	1.04	1.005–1.077
50-60	2	4.25	-	1.04	1.04–1.077
≥ 61	0	0	-	-	-
**Education level of patients with MGUS**					
Never been to school	5	4.16	120	-	-
primary	0	0	68	-	-
secondary	0	0	41	-	-
university	0	0	10	-	-
**Past medical history**					
No past medical history	4	1.74	230	-	-
Diabetes	1	3.33	3	-	-
Arterial hypertension	0	0	1	-	-
Nephropathy	0	0	1	-	-
Epilepsy	0	0	0	-	-
Others	0	0	0	-	-
**Obese**					
Yes	0	0	0	-	-
No	5	2.9	234	-	-
**Work station of the population**					
Pesticide storekeeper	0	0	40	-	-
Applicator	5	2.7	190	-	-
Supervisor	0	0	14	-	-
**Agricultural greenhouses**					
Yes	1	0.68	142	-	-
No	4	4.34	92	-	-
Time passed in the greenhouse (hour/day)	1.60†	-	4.99‡	-	-
**Agriculture type**					
Tomatoes	0	0	78	-	-
Citrus	4	4.34	92	-	-
Red fruits	1	1.62	62	-	-
**Days per year of treatment with pesticides**					
52 h	2	4.2	47	-	-
58 h	1	4.00	25	-	-
75 h	2	4.44	45	-	-
80 h	0	0	37	-	-
180 h	0	0	80	-	-
**Use of full protection equipments**					
Yes	4	3.41	117	-	-
No	1	1.88	53	-	-
Not adapted	0	0	64	-	-
**Cancer in first-degree relatives**					
No	5	2.9	234	-	-
Yes	0	0	0	-	-

Note: Student’s *t*-test, *p*-value < 0.05 indicates a statistically significant result.

MGUS, monoclonal gammopathy of undetermined signification; CI, confidence intervals; m ± s.d., mean ± standard deviation.

† , 95% CI: 2.84–6.04; ‡, 95% CI: 4.49–5.49.

The presence or absence of MGUS was found to be correlated with age, days per year of treatment with pesticides, time spent in the greenhouse, and education level of patients (Table 4). However, when all potential variables associated with the presence of MGUS were combined in a multiple stepwise conditional logistic regression analysis, it showed that the presence of MGUS was not significantly associated with any of the variables listed in Table 5.

**TABLE 4:** Correlation between the presence and the absence of MGUS with the different variables.

Variable	Presence or absence of MGUS	Age (years)	Days per year of treatment with pesticides (day)	Time passed in the green house (hour/day)
Age (years)	0.120*	-	-	-
Days per year of treatment with pesticides (day)	−0.118*	−0.238**	-	-
Time passed in the greenhouse (hour/day)	−0.125*	−0.438**	0.624**	-
Education level of patients	−0.125*	−0.308**	−0.346**	−0.085

MGUS, monoclonal gammopathy of undetermined signification; m ± s.d., mean ± standard deviation.

* , < 0.05;

** , *p*-value < 0.001.

**TABLE 5:** Comparison between the exposed and control groups.

Variable	Exposed (*n* = 239)				The non-exposed (*n* = 157)				*p*-value
	*n*	%	m ± s.d.	95% CI	*n*	%	m ± s.d.	95% CI	
Age in (years)	-	-	39.54 ± 11.51	38.07–41.01	-	-	49.19 ± 16.80	46.40–51.98	< 0.001
Total protein (g/L)	-	-	76.96 ± 3.63	76.23–77.15	-	-	75.19 ± 5.8	74.22–76.15	< 0.001
Albumin/Globulin Ratio	-	-	1.54 ± 0.27	1.5–1.57	-	-	1.4 ± 0.9	1.35–1.44	0.033
Albumin (g/L)	-	-	46.21 ± 3.32	45.78–46.63	-	-	43.38 ± 4.95	42.59–44.20	< 0.001
Prevalence of the monoclonal gammopathy in the exposed (*n* = 239) vs control (*n* = 157)	-	2.1	-	0.7–4.8	-	0.63	-	0.011–3.52	ns
Odds ratio of all the population	-	-	3.33	0.39–28.78	-	-	-	-	-
Prevalence of the monoclonal gammopathy in the exposed (*n* = 124) vs control (*n* = 110) older than 40 years old	-	4.03	-	1.49–9.62	-	0.91	-	0.16–4.97	< 0.001
Odds ratio with 95% CI for patients older than 40 years old	-	-	4.58	0.53–39.82	-	-	-	-	-
IgGλ	3	1.25	-	-	1	0.7	-	-	-
IgGκ	1	0.41	-	-	-	-	-	-	-
Biclonal IgGλ + IgGκ	1	0.41	-	-	-	-	-	-	-

Note: Student’s *t*-test, *p*-value < 0.05 indicates a statistically significant result.

MGUS, monoclonal gammopathy of undetermined signification; CI, Confidence intervals; m ± s.d., mean ± standard deviation; OR, odds ratio; IgG, immunoglobulin G; ns, not significant.

In Table 5, the comparison between the exposed and control groups, the mean age ± s.d. of the exposed group 39.54 ± 11.51 (95% CI: 38.07 – 41.01) was significantly lower (*p* < 0.001) than that of the control group 49.19 ± 16.80 (95% CI: 46.40 – 51.98). Although the patients in the exposed group were younger, the prevalence of MGUS was not significantly higher in the exposed group 2.1% (95% CI: 0.7% – 4.8%) compared to the control group 0.7% (95% CI: 0.01% – 3.9%). The prevalence of MGUS in the exposed group increased with age, with no cases reported before the age of 40. Comparing the prevalence in the exposed and control groups older than 40 years, the prevalence was significantly higher in the exposed groups 4.03% (95% CI: 1.49% – 9.62%) compared to the control groups 0.91% (95% CI: 0.16% – 4.97%). However, the OR was not significant 3.33 (95% CI: 0.39 – 28.78).

Finally, the characterisation of the monoclonal protein by serum immunofixation electrophoresis was as follows: IgG λ 3(1.25%), IgG κ 1(0.41%), and biclonal IgG κ +IgGκ1(0.41%) for the exposed group and IgG λ 1(0.7%) for the control group.

## Discussion

We found that the prevalence of MGUS was significantly higher in the exposed group older than 40 years 4.03% (95% CI: 1.49–9.62) compared to the control group 0.91% (95% CI: 0.16–4.97), with no cases reported before this age. However, the OR was not significant 3.33 (95% CI: 0.39–28.78), which can be explained by the size of the population and the mean age ± s.d. of the pesticide applicators of 39.54 ± 11.51. In contrast, the international studies discussed next found the same results with all recruited patients over 50 years of age.^19^ We sought for the association between pesticide exposure and MGUS in all age groups (from 18 years to 65 years old pesticide applicators). This choice was made at the start of the study.

Farming and exposure to pesticides are associated with the development of monoclonal gammopathies. A systematic review of 30 years of research on farming and MM, the pooled OR was for pesticide exposure 1.47 (95% CI: 1.11–1.94).^20^

Landgren et al., in a study of 555 male pesticide applicators older than 50 years, found that 38 were found to have MGUS, yielding a prevalence of 6.8% (95% CI: 5.0–9.3).^14^ In the Biomarkers of Exposure and Effect in Agriculture (BEEA) in the United States study, the estimated prevalence among men older than 50 years was 7.7% (95% CI: 6.3–9.1).^21^ In the predominantly white population of Olmsted Country, Minnesota, the estimated prevalence for the years 1995–2001 was 3.8% (95% CI: 3.4–4.2),^22^ and also in a population-based study of 12482 persons from the National Health and Nutrition Examination Survey (NHANES) for the years 1995–2004, the estimated prevalence was 2.8% (95% CI: 2.4–3.2).^23^

The AGRIculture and CANcer (AGRICAN) study is a large prospective cohort study. It includes farmers, farm workers and a significant proportion of women. The total number of participants is 180 000. Standardised incidence ratios (SIRs) and 95% CIs were calculated. The SIR was significantly higher for MM compared to the general population in men and women, SIR = 1.38, (95% CI: 1.18–1.62), and SIR = 1.26, (95% CI: 1.02–1.54), respectively.^11^ Landgren et al. found a 5.6-fold (95% CI: 1.9–16.6), 3.6-fold (95% CI: 1.5–10), and 2.6-fold (95% CI: 1.2–5.7) significantly increased risk of MGUS prevalence among users of the chlorinated insecticide dieldrin, the fumigant mixture carbon tetrachloride, and chlorothalonil, respectively.^14^ Three case-control studies in the United States and Canada were pooled into the North American Pooled Project (NAPP) to investigate associations between pesticide use and haematologic cancer risk using data from 547 MM cases and 2700 controls.^19^ Increased MM risk was observed forever use of *Carbaryl* (OR: 2.02, [95% CI: 1.28–3.21]), *Captan* (OR: 1.98, [95% CI: 1.04–3.77]) and *DDT* (*Dichloro-Diphenyl-Trichloroetane*) (OR: 1.44, [95% CI: 1.05–1.97]).^19^

All patients diagnosed with MGUS were illiterate and worked in the citrus crops; most of the time, they did not follow good practices for the use of pesticides. In fact, the citrus crop’s pesticide applicators were older and more senior in this function as pesticide applicators than the tomato and red fruit crop ones. Also, illiterate pesticide applicators lack knowledge regarding proper pesticide handling and do not respect the rules, such as smoking during pesticide handling. A lack of knowledge among pesticide applicators is the main reason for non-adherence to safety behaviours.

The pathogenesis of pesticide exposure and cancer remains unclear. The pathogenesis of monoclonal gammopathies and pesticide exposure may increase the production of oxygen-free radicals that damage deoxyribonucleic acid (DNA). Chromosomal aberrations and genotoxicity resulting from pesticide exposure may also contribute to the pathogenesis of multiple myeloma.^24^ Animal models have also shown that carbaryl exposure can modulate cancer risk through immunotoxic effects and imbalance of the immune response of T-helper cells (Th1/Th2).^25^ Chromosome abnormalities, including hyperdiploidy, translocations, deregulation of the retinoblastoma and cyclin D pathway, and deletion of chromosome 13, have been identified as contributing to the pathogenesis of MGUS.^26,27^

A small number of MGUS cases exhibited MM-associated somatic mutations, including KRAS, NRAS, DIS3, HIST1H1E, EGR1, and LTB. This suggests that the genomic landscape in these cases may be relatively simple.^28,29^ Moreover, circulating blood miRNA-744, miRNA-130a, miRNA-34a, let-7d, and let-7e were found to be dysregulated in both MGUS and MM. Notably, miRNA-34a and let-7e exhibited high sensitivity (91.1%) and specificity (96.7%) in distinguishing MGUS patients from healthy individuals.^30^

It has been demonstrated that common single nucleotide polymorphisms at 3p22.1 (rs1052501), 6p21.33 (rs2285803), 7p15.3 (rs4487645), and 17p11.2 (rs4273077) loci exert a significant influence on the risk of developing MM and MGUS.^16^

For the strengths of our study, we recruited pesticide applicators who have a higher exposure to pesticides than other agricultural workers. The pesticide applicators recruited had no risk factors; they were young (between 30 years and 50 years old), non-obese, caucasian people and had no family history of haematological disease.

The main limitations of our study were the size of the study sample because of the difficulty of recruiting this type of professional, with certain exposure to pesticides all year round, and that we chose to recruit all age groups, with a large proportion of participants between 30 years and 50 years old, even though we knew that this choice would have an impact on the prevalence. Our patients were exposed to mixtures of pesticides, making it impossible to determine the list of the most toxic pesticide molecules responsible for the genesis of MGUS.

## Conclusion

In this case-control study, we included in the exposed group among the agricultural workers those with proven occupational exposure to pesticides, namely the pesticide applicators. This study sheds light on the association of pesticide applicators’ occupational exposure to pesticides and MGUS, and the need to screen for monoclonal gammopathies from the age of 40 years instead of 50 years in the general population. We found a high prevalence of non-IgM MGUS in the exposed group compared to the control group, which is consistent with the results of several international studies from North America and Europe. In conclusion, we recommend screening for monoclonal gammopathies in pesticide applicators over 40 years of age in Morocco. Further targeted research studies with a larger number of pesticide applicators are needed to investigate the association between different synthetic pesticide molecules and their potential genotoxic effects with the development of MGUS in Moroccan young pesticide applicators.

### What is already known on this topic

- Several studies have reported an increase in the incidence of MM among farmers following pesticide use.
- Pesticide applicators have been shown to have more year-round exposure to pesticides than farmers.

### What this study adds

- The first North African study that sought an association between pesticide exposure and MGUS.
- Pesticide applicators have a proven exposure to pesticides.
- The prevalence of MGUS was 4.03% (95% CI: 1.49–9.62) in exposed patients older than 40-year-old.
